# Sensitivity Analysis of Reinforced Aluminum Based Metal Matrix Composites

**DOI:** 10.3390/ma15124225

**Published:** 2022-06-14

**Authors:** Fouzia Gillani, Muhammad Zubair Khan, Owaisur Rahman Shah

**Affiliations:** Department of Mechanical Engineering, Institute of Space Technology, Islamabad 44000, Pakistan; zubair_kj@yahoo.com (M.Z.K.); ors84@hotmail.com (O.R.S.)

**Keywords:** metal matrix composites, aluminum-based metal matrix composites, analysis of variance, energy dispersive spectroscopy, powder metallurgy, regression analysis, scanning electron microscope

## Abstract

Metal matrix composites (MMCs) have wide applications due to being lightweight, their high strength, and immense resistance to wear. To explore new generation materials like aluminum-based metal matrix composites (AMCs) for wide engineering applications, the present work aimed at investigating the effect of changes in composition, sintering time, and temperature on the hardness and surface roughness of AMCs containing SiC and ZrSiO_4_ in wt % of 5, 20, 30, and 40 binary and hybrid sample pallets. The samples have been prepared by powder metallurgy (PM) method under 1000 psi pressure. After compaction, the above pallets sintered at different temperatures ranging from 500 °C to 1100 °C with an increment of 200 °C and 15 min intervals for four levels of temperature and time, respectively. Afterwards, sensitivity analysis has been done by investigating the effect of chemical composition, sintering time, and sintering temperature of the binary and hybrid composites on hardness and surface roughness. Morphological studies on the composites were carried out using field emission scanning electron microscope (FESEM) with energy dispersive spectroscopy (EDS). It has been observed that hardness is increased by increasing the sintering temperature in the case of SiC, whereas surface roughness did not change much by changing the composition. Additionally, a rise in temperature lead to liquid-state sintering. SEM images obtained during the elemental analysis showed that porosity is generated within the samples after sintering due to the higher melting point of reinforcements compared to a base metal, i.e., aluminum. Mathematical equations have also been developed via regression analysis using Minitab and excel for the confirmation and validation of experimental data. Analysis of Variance (ANOVA) has also been done, and its tables are shown and discussed in the paper. Hence, the most optimized findings relating the changes in the composition of reinforcements, sintering temperature, and sintering time (input variables) with porosity, hardness, and surface roughness have been presented in the current study.

## 1. Introduction

Modern engineering materials, namely superalloys, shape memory alloys, ceramics, and fiber-reinforced composites, possess good thermal, chemical, physical, and mechanical properties, resulting in higher weight-to-volume ratio and better strength profiles. The needful properties of composites mainly depend upon the combined effect of fabrication techniques and reinforced/supplemented particles being added in some proportion. Many fabrication techniques are available and followed in the literature, such as spray stir casting, squeeze casting, compo-casting, powder metallurgy, pressure infiltration, and spray deposition method [1,2,3]. Bhoi et al. [4] concluded that production methods of MMCs can be categorized into two major categories: ex-situ and in-situ. The first synthesis route involves adding nano-reinforcements to the liquid or powdered metal. At the same time, the latter refers to methods that lead to the generation of ceramic nano-compounds by reaction during processing, such as using pressure infiltration/sintering. Among the preparation techniques of MMCs, the pressure infiltration process is one of the best suited for metal matrix syntactic foams. Higher infiltration pressure is preferred for perfect results [5,6,7,8]. Braszczyńska et al. [9] explored negative pressure infiltration methods to prepare AZ91 alloy matrix composites with fly ash cenospheres. Castro et al. [10] made an attractive combination of steel with a low density of foam prepared by pressure infiltration technology. This method utilizes inert gas (nitrogen/argon) pressure [11]. Orbulov [12,13] also prepared metal matrix syntactic foams (MMSFs) by using this method. After going through the exhaustive literature regarding the fabrication techniques and properties of aluminum matrix composites, Li et al. [14] found that the PM method is remarkably more beneficial as compared to other fabrication techniques like stir casting, squeeze casting, and compo casting, etc., due to low wettability produced between the molten aluminum matrix and the ceramic particle. El-Eskandarany [15] found that an additional advantage of PM is the uniform distribution of reinforcements. This uniform distribution not only improves the structural and mechanical properties. Kumar et al. [16] found that the MMCs preparation method and the type of supplements added play a critical role when machining. The conventional reinforcements used previously are SiC, Si_3_N_4_, Al-N, Al_2_O_3_, TiB_2_, ZrO_2_, and Y_2_O_3_. Along with the preparation technique, variation in grain structure during and after the preparation of MMCs largely affects the composites’ mechanical performance, which can be revealed by numerical and experimental evaluations [11]. AMCs are the most important and widely demanded composite which can be prepared via liquid and solid phase sintering, as Magabe et al. [17] prepared AMCs containing wt % 5, 10, 15, and 20 of Sn as a supplement to develop a new alloy named Al-Sn alloy into which wt % 5 of SiC was further added. The prepared AMCs were machined on wire electric discharge machining (WEDM), and it was observed that surface roughness increased by increasing the wt % of Sn.

Due to the presence of hard abrasive particles (reinforcements), which leads to high tool wear, the machinability of MMCs is very challenging. The behavior of such prepared MMCs before machining needs to be studied. Changes in wt % of reinforcements, sintering temperature, and compaction pressure may affect the MMCs behavior towards machining of MMCs [18]. A study of AMCs with SiC revealed that the compacting pressure and reinforcements percentage significantly affect the sintering temperature on microhardness and density. It is proven to achieve theoretical density near 95% for 10% reinforcement with 550 MPa sintered at 500 °C for 1 h [19]. Rana et al. [20] declared that Al-SiC has a high strength to weight ratio, which is three times more than mild steel, composites containing SiC as reinforcement have high modulus, strength values, high thermal stability, wear resistance, less weight, and a more effective load carrying capacity. Prasad et al. [21] also confirmed that this composite would exhibit good corrosion properties since SiC forms a preventive coating of silicon oxide at 1200 °C. In the last two decades, studies revealed that the wear behavior of aluminum-based metal matrix composites with different type of reinforcements is significantly affected by volume/weight fraction of reinforcement and particle size [22,23].

Pirkle et al. [24] found that SiC is known for its very high hardness and abrasion resistance. It is commonly meant for wear-intensive applications such as rollers and paper industry retainers. Therefore, Sadasivuni et al. [25] declared modern engineering composite materials as “engineered mechanical properties” incorporated in the form of reinforcements as micro or nano-sized particles.

The literature found that the addition of rigid ceramic reinforcements improves the wear resistance of composites as hard particles can behave like a load-bearing agent for the soft aluminum matrix. El-Kady et al. [26] reported that wear loss is significantly controlled by particle size. In hard micro-sized ceramic reinforcement deteriorates the ductility of composites, which can be controlled by adding nano-sized reinforcements [27]. Therefore, a combo of micro and nanoparticles as reinforcement could be a better option for developing new and advanced metal matrix composites [28].

A broad scope in significant areas of machining behavior and modeling of machining processes for MMCs has been offered. Further, to reduce the exhaustive experimentation for time and cost-effectiveness, some statistical and soft computing techniques are required to predict and optimize the process. There is a huge gap due to the unresolved issues while characterizing machining parameters and the accuracy achievement ratio of advanced materials [29,30]. Additionally, a limited study is present out of various available ceramics; ZrSiO_4_ is the least used as a supplementary material being added in the aluminum matrix. However, it is a better option, as fracture toughness and wears properties of ZrSiO_4_ are very high. Abdizadeh et al. [31] found that fabrication of ZrSiO_4_ reinforced MMCs using different techniques is an excellent option to attain better mechanical properties. Limited literature is available on aluminum composites involving both silicon carbide and ZrSiO_4_ reinforcements combined to form hybrid AMCs. It is exciting and provides scope to study harsh tribological challenges. Therefore, the current research aims to investigate the mechanical properties of AMCs using SiC and ZrSiO_4_ as reinforcements. The results obtained showed exciting facts relating to changes in the composition of reinforcements and their sintering temperature and time.

## 2. Experimental Setup

The composites have been fabricated through the powder metallurgy process, which involves various stages, including measurement of elemental powders, mixing of powders, compaction, and sintering of the compact profiles. Sintering temperature, variation in wt % of supplementary powders and sintering time were the input experimental parameters. The response of these variables was checked for hardness and surface roughness of the sintered samples. Morphological studies on the composites were carried out using FESEM (manufactured by TESCAN, Brno, Czech Republic) which generate ultra-high resolution imaging at low accelerating voltages and small working distances. KeV mentioned in images is electron volt, which is the measure of an amount of kinetic energy gained by a single electron. The first step in preparing the samples was to weigh the required compositions. Powders of metal and reinforcements were blended using a ball mill with each reinforcement. A centrifugal ball mill thoroughly mixed the calculated ratio of soft aluminum powder and ceramic particles. To get uniform mixing, 10:1 ball to powder ratio (BPR) was maintained throughout mixing. The mixing speed and time were 150 rpm and 20 min, respectively.

**Figure 1:**
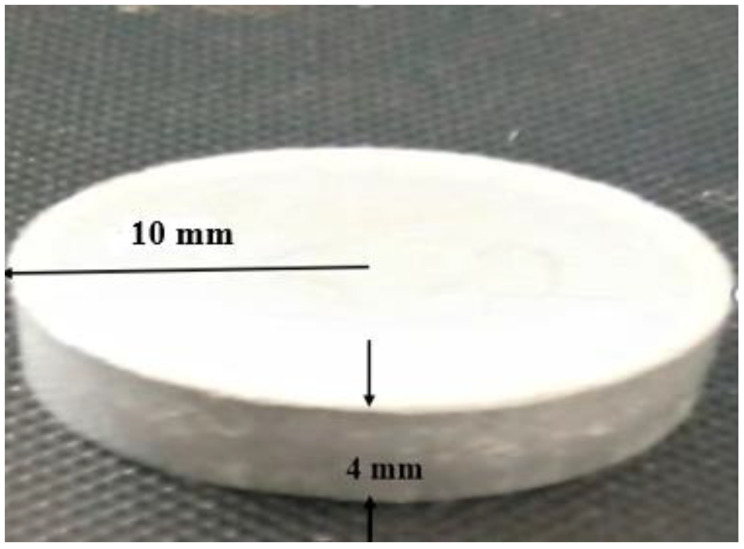
Sample pallets.

Uniformly mixed powders were then compacted press into a ‘green form’, thus obtaining approximately 80% dense by using a uniaxial hydraulic pallet press machine to compact the powder by using 1000 psi pressure for two minutes holding time. A prepared sample of 4 mm thickness and 10 mm radius is shown below in Figure 1.

After compaction of powders into pallets, the samples were sintered by heating them to the desired processing temperature in the furnace. After sintering, the samples were furnace cooled. The fabricated samples were then tested for hardness, surface roughness, and morphological studies. Hardness of sintered samples was measured using a Rockwell hardness tester using a load of 100 Kg for 7 s. The surface roughness of the sintered samples was measured using a portable roughness meter known as surftest SJ-210 [mm] with detector measuring force of 0.75 mN and stylus tip (diamond tip) angle of 60°. It can measure the range up to 360 µm. Three samples of each composition were prepared and tested. Theoretical densities of the composite specimens were determined using a high-precision digital electronic weighing balance with an accuracy of 0.01 mg.

### 2.1. Chemical Compositions of MMCs

The aluminum powder taken for each sample was 3.108 g. The reinforcement percentages are given below in Table 1.

### 2.2. Mixing and Compaction of Powders

The measured loose powders were mixed according to the proposed composition and ball milled again for proper mixing. Ball milled and measured powders were compacted via a pneumatic, hydraulic press with 1000 psi pressure for 2 min holding time. The sample is of the circular shape of a 1-inch diameter. Twelve binary and hybrid samples of the above-mentioned compositions have been prepared. We identified the samples as S1, S2, and S3 up to S12, as mentioned in Table 2.

### 2.3. Sintering

Sintering can be done as solid-state sintering and liquid phase sintering. We have implemented solid-phase sintering in a vacuum furnace [4]. Sintering is done by heating the green body of binary and hybrid samples prepared by PM to the desired processing temperature in the furnace. After sintering, the samples were furnace cooled. The sintering temperatures and time for different compositions of MMCs are described below.

## 3. Experimental Results

It is observed that sintering consolidation is near to 100% during the molten phase and agglomeration started, so it is needed to check at which temperature consolidation happens. Mathematical equations were estimated for each input parameter using regression analysis to validate the experimental results. Here Table 3. Showed the roughness and hardness results against each sample.

### 3.1. EDS Analysis of Samples

**Figure 2:**
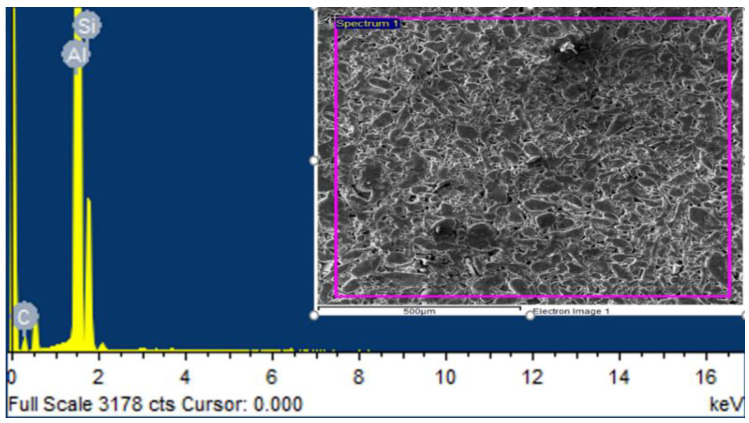
EDS spectrum of Al + 5%SiC reinforced composites.

EDS analysis has evaluated the particles’ presence and weight percentage in sintered AMCs [32,33,34]. Peaks in the graph show the wt % of each element [35]. Here, EDS analysis of the samples containing 5% by weight of SiC as shown in Figure 2. estimated that SiC particles are present in the synthesized composites. Table 4. showed the elements along with their wt % and atomic%.

**Figure 3:**
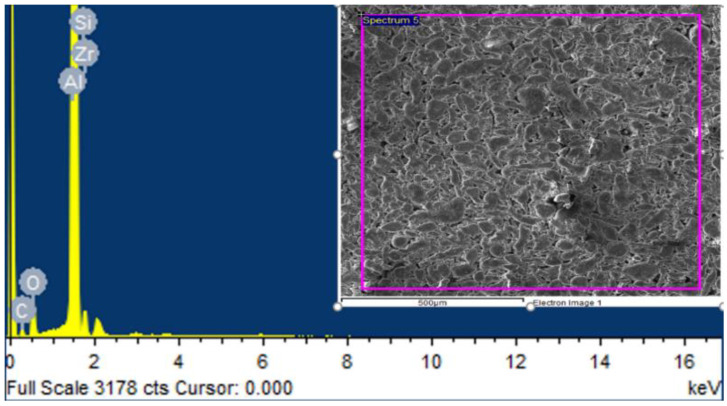
EDS spectrum of Al + 5%ZrSiO_4_ reinforced composites.

Similarly, EDS analysis of the sample containing 5% by weight of ZrSiO_4_ in Figure 3. predicted that ZrSiO_4_ particles are present in the synthesized composite of Al-ZrSiO_4_. Table 5. showed the elements along with their wt % and atomic%.

### 3.2. Density and Porosity

**Figure 4:**
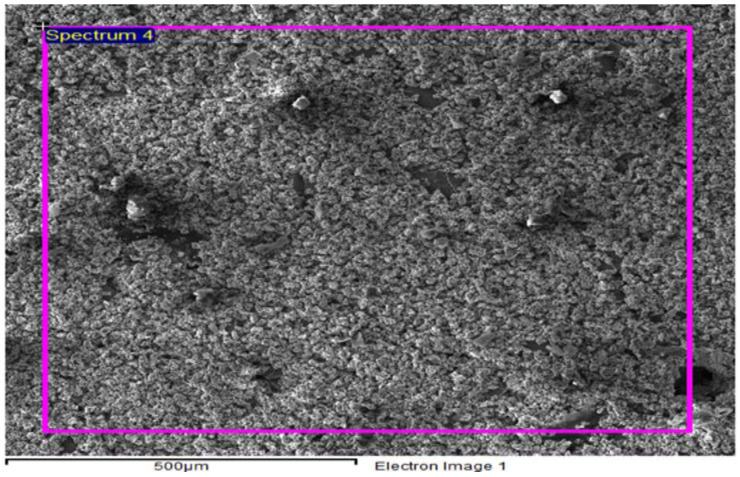
SEM image of Al + 40% SiC sintered at 1100 °C showing agglomeration of particles and porosity.

Theocratical measurement of density revealed that it is possible to attain 98% of the density (theoretical) for samples of 20% reinforcement compact with 1000 Mpa sintered at 700 °C. It has been observed that compaction pressure and reinforcement wt % have a higher impact on density and porosity than sintering temperature. Figure 4 showed agglomeration and porosity in sample containing 40% SiC sintered at 1100 °C.

### 3.3. Hardness of Sintered Samples

The hardness of each composition was measured for three similar samples. It is observed from the above table that the Al-SiC matrix with wt % 5 and 40 have the same and maximum hardness, i.e., 79 HRA out of all other samples of Al-SiC. The results can observe that as temperature increases, the hardness increases, whereas at elevated temperature, concentration does not impart much in the case of Al-SiC samples. However, in the case of ZrSiO_4_, it is also important to note that maximum concentration of ZrSiO_4_ imparts greater hardness to AMCs at low temperature and higher sintering time than at high temperature and lower sintering time. Therefore, concentration of reinforcements (wt %) contributes significantly towards hardness in the case of Al-ZrSiO_4_.

### 3.4. Regression Analysis for Hardness of Sintered Samples

**Figure 5:**
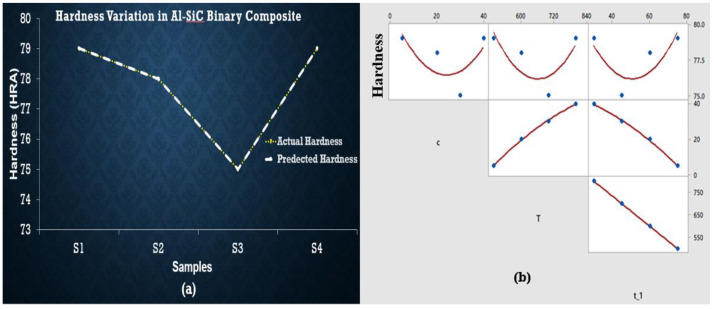
(**a**) Regression plot for Al-SiC showing overlapping of actual and predicted hardness values points (yellow dotted line presents actual value of hardness and white line shows predicted values). (**b**) Matrix plot showing the individual relationship between hardness and input variables for Al-SiC.

**Figure 6:**
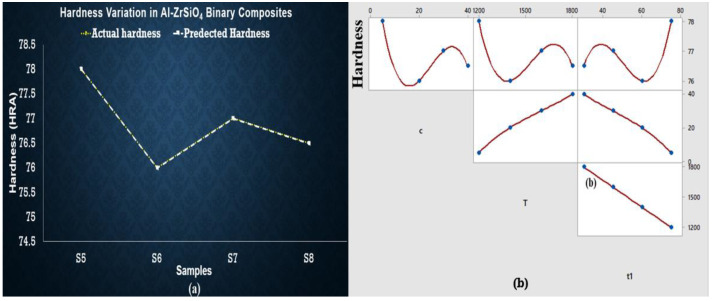
(**a**) Regression plot for Al-ZrSiO_4_ showing overlapping of actual and predicted hardness values points (yellow dotted line presents actual value of hardness and white line shows predicted values). (**b**) Matrix plot showing the individual relationship between hardness and input variables for Al-ZrSiO_4_.

Experimental results show that from samples S1 to S4, the temperature and concentration of SiC are being increased. At the same time, hardness decreases from S1 to S3 and then increases up to S4; Equation (1) shows the same behavior, but the effect of concentration is negative, and the effect of temperature is favorable. Still, overall hardness is also dependent on intercept terms. No one from an independent variable is changed indecently, and their combined effect shows the same behavior as experimental results shown by the predicted hardness line in Figure 5. Moreover, maximum hardness was observed for S1 and S4. Similarly, for S5 to S8 the predicted and actual hardness overlapped as shown in Figure 6.

Hardness = 52.5 + 0.058 T − 0.53c_1_ (1)

Hardness = 6.375 + 0.7875c_1_ + 0.06313T − 0.007500c_1_ × c_1_ (2)

Hardness = −79.33 − 5.200 c_1_ + 1.400 c_2_ + 0.2567 T (3)

where c_1_ is composition of Al-SiC samples, and c_2_ is the composition of Al-ZrSiO_4_ samples and T sintering temperature.

**Figure 7:**
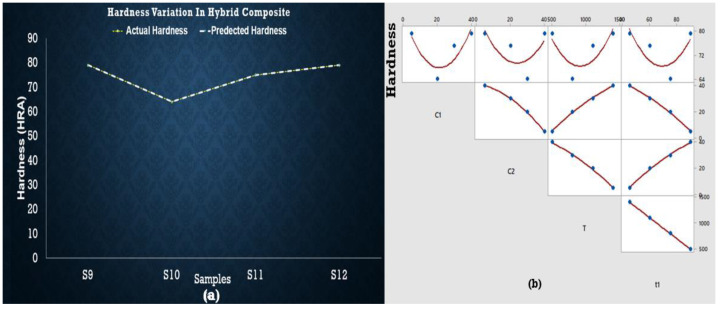
(**a**) Regression plot for Al-SiC-ZrSiO_4_ showing overlapping of actual and predicted hardness values points (yellow dotted line presents actual value of hardness and white line shows predicted values). (**b**) Matrix plot showing the individual relationship between hardness and input variables for Al-SiC-ZrSiO_4_.

Equation (3) was developed to estimate the relations between experimental results, which shows that c_1_ (composition of SiC) decreases the hardness. In contrast, c_2_ (composition of ZrSiO_4_) and sintering temperature tend to increase the hardness when all independent variables are changed. The maximum hardness was observed at S9 (at minimum temperature and concentration) and S12 (at maximum temperature and concentration) as shown in Figure 7. S10 represents the smallest value of hardness, whereas the regression equations do not entirely fulfill the statistical requirements observed in ANOVA results and are presented in a supplementary file. As there is no significant factor (*p* < 0.05) found in the case of hardness, their behavior is the best representation of experimental data.

### 3.5. Roughness of Sintered Samples

**Figure 8:**
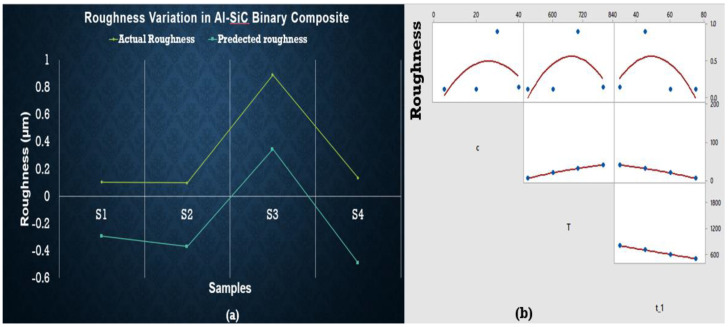
(**a**) Regression plot for Al-SiC showing overlapping of actual and predicted roughness values points (yellow line presents actual value of hardness and blue line shows predicted values). (**b**) Matrix plot showing the individual relationship between roughness and input variables for Al-SiC.

Surface Roughness was measured by using a portable roughness meter known as surftest SJ-210 [mm] with detector measuring force of 0.75 mN and stylus tip (diamond tip) angle of 60°. It can measure the range up to 360 µm. The result for Al-SiC AMCs shows that composition is not significantly affecting the surface roughness at elevated temperatures. Still, temperature and composition do have a combined negative effect on it. Additionally, there is another parameter for the behavior shown in Figure 8. for surface roughness. This is verified by the regression equations given below. Only S3 shows the maximum value of roughness.

### 3.6. Regression Analysis for Roughness of Sintered Samples

**Figure 9:**
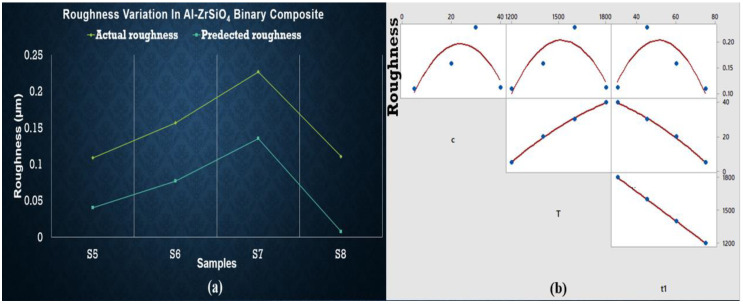
(**a**) Regression plot for Al-ZrSiO_4_ showing overlapping of actual and predicted roughness values points (yellow line presents actual value of hardness and blue line shows predicted values). (**b**) Matrix plot showing the individual relationship between roughness and input variables for Al-ZrSiO_4_.

The intercept value is high in Equation (4), which shows that some other parameter is involved while deciding the surface roughness of said samples. The c_1_*T term also has a minor influence on reducing roughness. In comparison, ANOVA results observed no significant factor. Equation (5) presents the effect of change in composition with the sintering temperature, which can be observed in the graphical representation in Figure 9. The roughness of ZrSiO_4_ samples is minimum for S5 (at minimum temperature and concentration) and S8 (at maximum temperature and concentration). S7 shows maximum roughness, which is undesirable. Surface roughness is increased by c_1_ while c_2_ and sintering temperature tends to decrease it.

Roughness = −40.45 − 0.08175 c_1_ + 0.08579 T − 0.000775 c_1_ × T (4)

Roughness = −4.598 − 0.02765 c_1_ + 0.004057 T − 0.000930 c_2_ × c_2_ (5)

Roughness = 5.714 + 0.1128 c_1_ − 0.06960 c_2_ − 0.006910 (6)

where c_1_ is the composition of Al-SiC samples and c_2_ is the composition of Al-ZrSiO_4_ samples and T is the sintering temperature.

**Figure 10:**
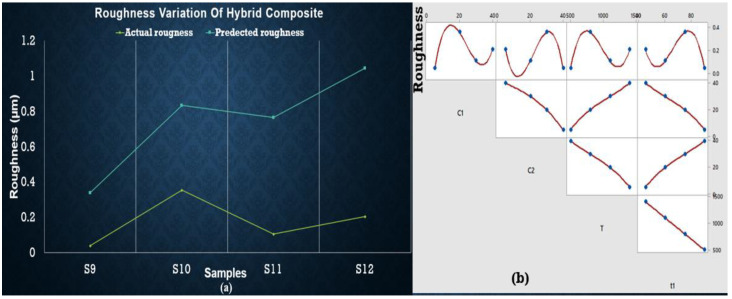
(**a**) Regression plot for Al-SiC-ZrSiO_4_ showing overlapping of actual and predicted roughness values points (yellow line presents actual value of hardness and blue line shows predicted values). (**b**) Matrix plot showing the individual relationship between roughness and input variables for Al-SiC-ZrSiO_4_.

The maximum value for roughness was observed for S12 based on experimental data, while predicted data based on the regression equation shows the maximum roughness value for S10. It can be concluded that S9 (at the lowest concentration and temperature) has minimum roughness value as shown in Figure 10. Again, ANOVA results are presented in a supplementary file that shows no variable being significantly affecting the roughness.

## 4. Discussion

Porosity originated in the samples due to the difference between melting points of base metal and reinforcements. As soon as the difference increases, porosity increases. This is also justified in the previous studies—that increment of reinforcements drastically grows the porosity because of the clustering and agglomeration at high concentration of particles, dense, random distribution, and a high melting point of reinforcement particles hinders the effective sintering operation [26,36,37]. Hence, reinforcement particles act as a barrier to the rearrangement and diffusion of particles, leading to higher porosity irrespective of the sintering temperature [38,39]. As far as hardness is concerned, it is clear from graphical representation that S5 to S8 temperature and concentration are increased but maximum hardness is observed at S5, then it decreases up to S6, increases for S7, and finally decreases for S8 as well. So, S5 shows the best results for hardness. Hybrid AMCs showed similar hardness at two different concentrations: temperature and time. AMCs with SiC wt % 5, ZrSiO_4_ wt % 40 and SiC with wt % 40, ZrSiO_4_ having wt % 5 shows the same hardness sintered at 500 °C (60 min) and 1100 °C (15 min) respectively. Therefore, all the above results showed that hardness is greatly influenced by sintering time, temperature, and concentration of reinforcements. It is evident from the above plots that the predicted and actual hardness lies on one another. The addition of SiC in the aluminum matrix increases the porosity of the composite. The increase in the porosity can be attributed to the high hardness of SiC, which hinders the proper compaction of composite powders [40]. The increased porosity of Al-SiC composites has also been reported by other researchers [41,42,43]. In the case of ZrSiO_4_, maximum hardness was observed at low sintering temperature and the least reinforcement concentration for maximum time. This is due to the reason that ZrSiO_4_ has a very high melting temperature, nearly 2550 °C. The regression Equation (2) did not wholly satisfy the statistical requirements obtained in ANOVA results (shown in supplementary file), as there is no significant factor (*p* < 0.05) found in the case of hardness, but their behavior is the best representation of experimental data, especially in the case of predicted hardness values by these equations which give us some points similar to those of experimental values. The enhanced hardness of the composites may be due to the uniform distribution of SiC and ZrSiO_4_ in the composites and the high density of ZrSiO_4_. Actual values of roughness in the case of SiC and ZrSiO_4_ samples are the same as the predicted roughness. Predicted and actual values follow the same behavior but did not lie on one another, whereas actual roughness behavior of hybrid samples deviates from the predicted values. In the future, this study can be extended by making a comparison between morphology of the initial powders and processed ones, and machining of the prepared samples (in the current study) using non-conventional machining operations. Optimization of machining parameters of machining such composites also covers the currently needed area to explore.

## 5. Conclusions

In the experimental work, Al-SiC, Al-ZrSiO_4_, and hybrid metal matrix composites of Al-SiC-ZrSiO_4_ were prepared with four different compositions sintered at four different sintering temperatures together with four different time frames using the powder metallurgy method. The important conclusions are:

- EDS analysis validate the presence of SiC and ZrSiO_4_ in the fabricated composites, as the distribution of reinforced particles in the aluminum matrix is estimated from elemental analysis.
- It is concluded from the current study that the hardness of aluminum is enhanced the most with the addition of maximum wt % of SiC, i.e., wt % 40 at elevated temperature in the case of binary and hybrid MMCs.
- Enhancement of roughness is low in the case of less wt % of SiC but higher wt % of ZrSiO_4_.
- It has also been observed that the intercept values are higher in the case of hardness and roughness of Al-SiC binary samples. However, for hybrid samples, some parameters other than after composition, temperature, and time are also responsible for this behavior, which must be studied in further detail.
- It is concluded from the current study that the sintering time did not impart significant hardness and roughness in all the materials that were studied.
- Future studies may focus on investigating machining parameters adopted for the non-conventional machining of these samples. Effects of machining on the hardness and surface roughness of these samples and their parametric comparison before and after machining must be an exciting area to explore.

## Figures and Tables

**Table 1:** Designed ratio of wt % of Al-powder and reinforcements in MMCs.

Sample	Type	SiC (37–45 µm) (wt %)	ZrSiO_4_ (<50 nm) (wt %)	SiC + ZrSiO_4_ (wt %)
Al-SiC	Binary	5	-	-
		20	-	-
		30	-	-
		40	-	-
Al-ZrSiO_4_	Binary	-	5	-
		-	20	-
		-	30	-
		-	40	-
Al-SiC-ZrSiO_4_	Hybrid	5	40	5 + 40
		20	30	20 + 30
		30	20	30 + 20
		40	5	40 + 5

**Table 2:** Sintering temperature and sintering time for the samples.

Sample	Type	Sample	SiC (37–45 µm) (wt %)	ZrSiO_4_ (<50 nm) (wt %)	SiC + ZrSiO_4_ (wt %)	Sintering Temperature (°C)	Sintering Time (min)
Al-SiC	Binary	S1	5	-	-	500	60
		S2	20	-	-	700	45
		S3	30	-	-	900	30
		S4	40	-	-	1100	15
Al-ZrSiO_4_	Binary	S5	-	5	-	500	60
		S6	-	20	-	700	45
		S7	-	30	-	900	30
		S8	-	40	-	1100	15
Al-SiC-ZrSiO_4_	Hybrid	S9	5	40	5 + 40	500	60
		S10	20	30	20 + 30	700	45
		S11	30	20	30 + 20	900	30
		S12	40	5	40 + 5	1100	15

**Table 3:** Results of hardness and roughness.

Sample	Type	Sample	Mean Hardness (HRA) ± Std	Roughness (µm)
Al-SiC	Binary	S1	79.0 ± 3.6	0.104
		S2	78.0 ± 3.3	0.099
		S3	75.0 ± 4.1	0.89
		S4	79.0 ± 3.6	0.132
Al-ZrSiO_4_	Binary	S5	78.0 ± 3.3	0.109
		S6	76.0 ± 4.2	0.157
		S7	77.0 ± 3.5	0.227
		S8	77.0 ± 3.5	0.111
Al-SiC-ZrSiO_4_	Hybrid	S9	79.0 ± 3.6	0.039
		S10	64.0 ± 2.5	0.354
		S11	75.0 ± 4.1	0.105
		S12	79.0 ± 3.6	0.204

**Table 4:** List of elements found in the Al + 5%SiC reinforced composites.

Elements	Weight%	Atomic%
Al	75.3	61.7
Si	6.8	5.3
C	17.9	33.0

**Table 5:** List of elements found in the Al + 5%ZrSiO_4_ reinforced composites.

Elements	Weight%	Atomic%
Al	71.7	61.6
Si	2.5	2.0
C	9.8	19.0
O	11.2	16.3
Zr	4.8	1.2

## Data Availability

Not applicable.

## Supplementary Materials

The following supporting information can be downloaded at: https://www.mdpi.com/article/10.3390/ma15124225/s1.
