# Supplementary material for: Sensitivity Analysis of Reinforced Aluminum Based Metal Matrix Composites
Source: Materials (Basel). 2022 Jun 14;15(12):4225. doi: 10.3390/ma15124225 (PMC9227862; doi:10.3390/ma15124225)
Supplement: Supplementary file 1 [file materials-15-04225-s001.zip › materials-1756987-supplementary.pdf]

ANOVA results for hardness

Al-SiC hardness

Statistical analysis for hardness of AMCs reinforced with SiC

Regression Analysis: Hardness versus C1, T, t
The following terms cannot be estimated and were removed:
ts
Method

Rows unused 1
Table S1: Analysis of Variance.

Source	DF	Adj SS	Adj MS	F-Value	P-Value
Regression	2	2.583	1.292	0.16	0.872
C1	1	2.133	2.133	0.26	0.699
T	1	1.908	1.908	0.23	0.713
Error	1	8.167	8.167		
Total	3	10.750			

Model Summary

S	R-sq	R-sq(adj)	R-sq(pred)
2.85774	24.03%	0.00%	*

“*” in a regression table indicate the level of the statistical significance of a regression coefficient.

Table S2: Coefficients.

Term	Coef	SE Coef	T-Value	P-Value	VIF
Constant	52.5	53.9	0.97	0.508	
C1	-0.53	1.04	-0.51	0.699	89.17
T	0.058	0.121	0.48	0.713	89.17

Regression Equation

Hardness = 52.5 - 0.53 C1 + 0.058 T

Fits and Diagnostics for Unusual Observations

Obs	Hardness	Fit	Resid	Std Resid	
2	79.00	79.00	0.00	*	X

X Unusual X

“*” in a regression table indicate the level of the statistical significance of a regression coefficient.

Al-$ZrSiO_4$ composite hardness

Statistical analysis for hardness of AMCs reinforced with $ZrSiO_4$

Regression Analysis: Hardness versus C1, T, ts
The following terms cannot be estimated and were removed:
ts
Method

Rows unused 1

Table S3: Analysis of Variance

Source	DF	Adj SS	Adj MS	F-Value	P-Value
Regression	3	2.18750	0.7292	*	*
C1	1	1.16189	1.1619	*	*
T	1	0.77799	0.7780	*	*
C1*C1	1	0.37500	0.3750	*	*
Error	0	0.00000	*		
Total	3	2.18750			

Model Summary

S	R-sq	R-sq(adj)	R-sq(pred)
*	100.00%	*	*

"*" in a regression table indicate the level of the statistical significance of a regression coefficient.

Table S4: Coefficients

Term	Coef	SE Coef	T-Value	P-Value	VIF
Constant	6.375	*	*	*	
C1	-0.7875	*	*	*	356.95
T	0.06313	*	*	*	1024.37
C1*C1	-0.007500	*	*	*	208.76

"*" in a regression table indicate the level of the statistical significance of a regression coefficient.

Regression Equation

Hardness = 6.375 - 0.7875 C1 + 0.06313 T - 0.007500 C1*C1

*** NOTE * Could not graph the specified residual type because MSE = 0 or the degrees of freedom for error = 0.**

Regression Analysis: Hardness versus C1, T, ts

The following terms cannot be estimated and were removed:

ts

Method

Rows unused 1

Table S5: Analysis of Variance

Source	DF	Adj SS	Adj MS	F-Value	P-Value
Regression	2	1.8125	0.9062	2.42	0.414
C1	1	1.2000	1.2000	3.20	0.325
T	1	1.0129	1.0129	2.70	0.348
Error	1	0.3750	0.3750		
Total	3	2.1875			

Model Summary

S	R-sq	R-sq(adj)	R-sq(pred)
0.612372	82.86%	48.57%	*

“*” in a regression table indicate the level of the statistical significance of a regression coefficient.

Table S6: Coefficients

Term	Coef	SE Coef	T-Value	P-Value	VIF
Constant	54.5	14.1	3.86	0.161	
C1	-0.400	0.224	-1.79	0.325	89.17
T	0.0212	0.0129	1.64	0.348	89.17

Regression Equation

Hardness = 54.5 - 0.400 C1 + 0.0212 T

Fits and Diagnostics for Unusual Observations

Obs	Hardness	Fit	Resid	Std Resid	
2	78.000	78.000	0.000	*	X

X Unusual X

“*” in a regression table indicate the level of the statistical significance of a regression coefficient.

Al-SiC-$ZrSiO_4$ hybrid composite hardness

Statistical analysis for hardness of AMCs reinforced with SiC and $ZrSiO_4$

Regression Analysis: Hardness versus c1, c2, T, t1
The following terms cannot be estimated and were removed:
t1
Table S7: Analysis of Variance

Source	DF	Adj SS	Adj MS	F-Value	P-Value
Regression	3	150.750	50.250	*	*
c1	1	112.667	112.667	*	*
c2	1	8.167	8.167	*	*
T	1	55.934	55.934	*	*
Error	0	0.000	*		
Total	3	150.750			

Model Summary

S	R-sq	R-sq(adj)	R-sq(pred)
*	100.00%	*	*

"*" in a regression table indicate the level of the statistical significance of a regression coefficient.

Table S8: Coefficients

Term	Coef	SE Coef	T-Value	P-Value	VIF
Constant	-79.33	*	*	*	
c1	-5.200	*	*	*	160.50
c2	1.400	*	*	*	160.50
T	0.2567	*	*	*	530.00

"*" in a regression table indicate the level of the statistical significance of a regression coefficient.
Regression Equation

Hardness = -79.33 - 5.200 c1 + 1.400 c2 + 0.2567 T

ANOVA results for roughness

Statistical analysis for surface roughness of AMCs reinforced with SiC

Regression Analysis: roughness versus c1, T, t1
The following terms cannot be estimated and were removed:
t1
Table S9: Analysis of Variance

Source	DF	Adj SS	Adj MS	F-Value	P-Value
Regression	2	0.05508	0.02754	0.07	0.938
c1	1	0.01680	0.01680	0.04	0.871
T	1	0.01170	0.01170	0.03	0.892
Error	1	0.39990	0.39990		
Total	3	0.45498			

Model Summary

S	R-sq	R-sq(adj)	R-sq(pred)
0.632377	12.11%	0.00%	*

"*" in a regression table indicate the level of the statistical significance of a regression coefficient.

Table S10: Coefficients

Term	Coef	SE Coef	T-Value	P-Value	VIF
Constant	2.2	11.9	0.18	0.886	
c1	0.047	0.231	0.20	0.871	89.17
T	-0.0046	0.0267	-0.17	0.892	89.17

Regression Equation

roughness = 2.2 + 0.047 c1 - 0.0046 T

Fits and Diagnostics for Unusual Observations

Obs	roughness	Fit	Resid	Std Resid	
1	0.104	0.104	0.000	*	X

X Unusual X

"*" in a regression table indicate the level of the statistical significance of a regression coefficient.

Al-SiC roughness

Regression Analysis: roughness versus c1, T, t1

The following terms cannot be estimated and were removed:
t1
Table S11: Analysis of Variance

Source	DF	Adj SS	Adj MS	F-Value	P-Value
Regression	3	0.454985	0.15166	*	*
c1	1	0.038189	0.03819	*	*
T	1	0.331528	0.33153	*	*
c1*T	1	0.399900	0.39990	*	*
Error	0	0.000000	*		
Total	3	0.454985			

Model Summary

S	R-sq	R-sq(adj)	R-sq(pred)
*	100.00%	*	*

"*" in a regression table indicate the level of the statistical significance of a regression coefficient.
Table S12: Coefficients

Term	Coef	SE Coef	T-Value	P-Value	VIF
Constant	-40.45	*	*	*	
c1	-0.08175	*	*	*	117.03
T	0.08579	*	*	*	1110.00
c1*T	-0.000775	*	*	*	714.28

Regression Equation

roughness = -40.45 - 0.08175 c1 + 0.08579 T - 0.000775 c1*T
"*" in a regression table indicate the level of the statistical significance of a regression coefficient.

Statistical analysis for surface roughness of AMCs reinforced with $ZrSiO_4$

Regression Analysis: roughness versus c1, T, t1
The following terms cannot be estimated and were removed:
t1
Table S13: Analysis of Variance

Source	DF	Adj SS	Adj MS	F-Value	P-Value
Regression	2	0.003410	0.001705	0.30	0.793
c1	1	0.003121	0.003121	0.54	0.596
T	1	0.002889	0.002889	0.50	0.608
Error	1	0.005766	0.005766		

Total	3	0.009176

Model Summary

S	R-sq	R-sq(adj)	R-sq(pred)
0.0759342	37.16%	0.00%	*

"*" in a regression table indicate the level of the statistical significance of a regression coefficient.

Table S14: Coefficients

Term	Coef	SE Coef	T-Value	P-Value	VIF
Constant	1.37	1.75	0.78	0.578	
c1	0.0204	0.0277	0.74	0.596	89.17
T	-0.00113	0.00160	-0.71	0.608	89.17

Regression Equation

roughness = 1.37 + 0.0204 c1 - 0.00113 T

Fits and Diagnostics for Unusual Observations

Obs	rougness	Fit	Resid	Std Resid	
1	0.1090	0.1090	0.0000	*	X

X Unusual X

"*" in a regression table indicate the level of the statistical significance of a regression coefficient.

Al-ZrSiO$_4$ roughness

Regression Analysis: roughness versus c1, T, t1

The following terms cannot be estimated and were removed:
t1

Table S15: Analysis of Variance

Source	DF	Adj SS	Adj MS	F-Value	P-Value
Regression	3	0.009176	0.003059	*	*
c1	1	0.001432	0.001432	*	*
T	1	0.003214	0.003214	*	*
c1*c1	1	0.005766	0.005766	*	*
Error	0	0.000000	*		
Total	3	0.009176			

Model Summary

S	R-sq	R-sq(adj)	R-sq(pred)
*	100.00%	*	*

“*” in a regression table indicate the level of the statistical significance of a regression coefficient.
Table S16: Coefficients

Term	Coef	SE Coef	T-Value	P-Value	VIF
Constant	-4.598	*	*	*	
c1	-0.02765	*	*	*	356.95
T	0.004057	*	*	*	1024.37
c1*c1	-0.000930	*	*	*	208.76

Regression Equation

roughness = -4.598 - 0.02765 c1 + 0.004057 T - 0.000930 c1*c1
“*” in a regression table indicate the level of the statistical significance of a regression coefficient.
Hybrid composite roughness

Statistical analysis for surface roughness of AMCs reinforced with SiC and $ZrSiO_4$

Regression Analysis: roughness versus c1, c2, T, t1
The following terms cannot be estimated and were removed:
t1, c1*c2
Table S17: Analysis of Variance

Source	DF	Adj SS	Adj MS	F-Value	P-Value
Regression	3	0.056277	0.01876	*	*
c1	1	0.053016	0.05302	*	*
c2	1	0.020184	0.02018	*	*
T	1	0.040541	0.04054	*	*
Error	0	0.000000	*		
Total	3	0.056277			

Model Summary

S	R-sq	R-sq(adj)	R-sq(pred)
*	100.00%	*	*

“*” in a regression table indicate the level of the statistical significance of a regression coefficient.
Table S18: Coefficients

Term	Coef	SE Coef	T-Value	P-Value	VIF
Constant	5.714	*	*	*	
c1	0.1128	*	*	*	160.50

c2	-0.06960	*	*	*	160.50
T	-0.006910	*	*	*	530.00

Regression Equation

roughness = 5.714 + 0.1128 c1 - 0.06960 c2 - 0.006910 T

"*" in a regression table indicate the level of the statistical significance of a regression coefficient.

<u>SEM Images of Al-SiC wt% 5, 20, 30 & 40</u>

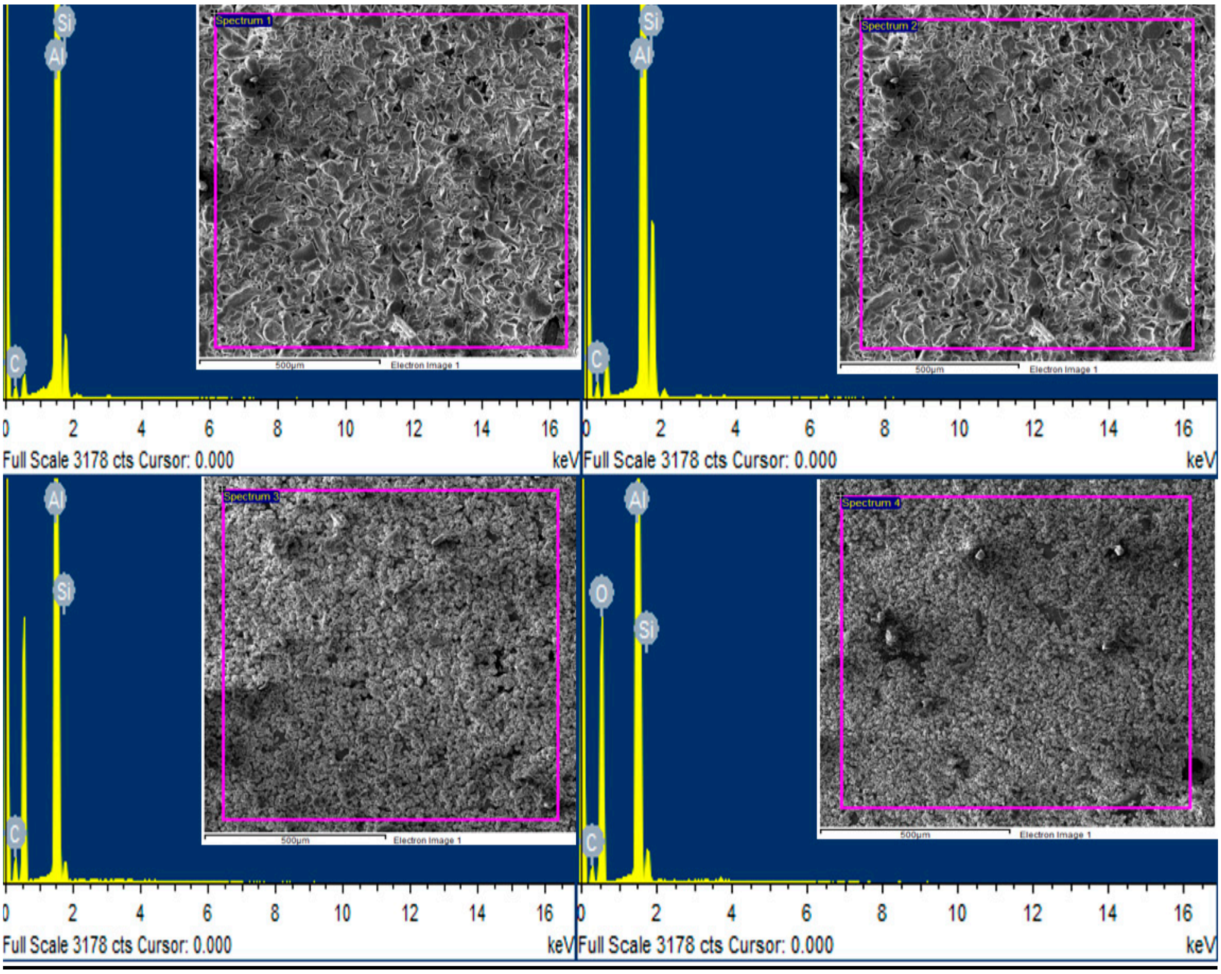

Figure S1. EDS Spectra of Al-SiC reinforced binary composites with wt % of 5, 20, 30 and 40 from left to right in the row sequency.

SEM Images of Al- $ZrSiO_4$ wt% 5, 20, 30 & 40

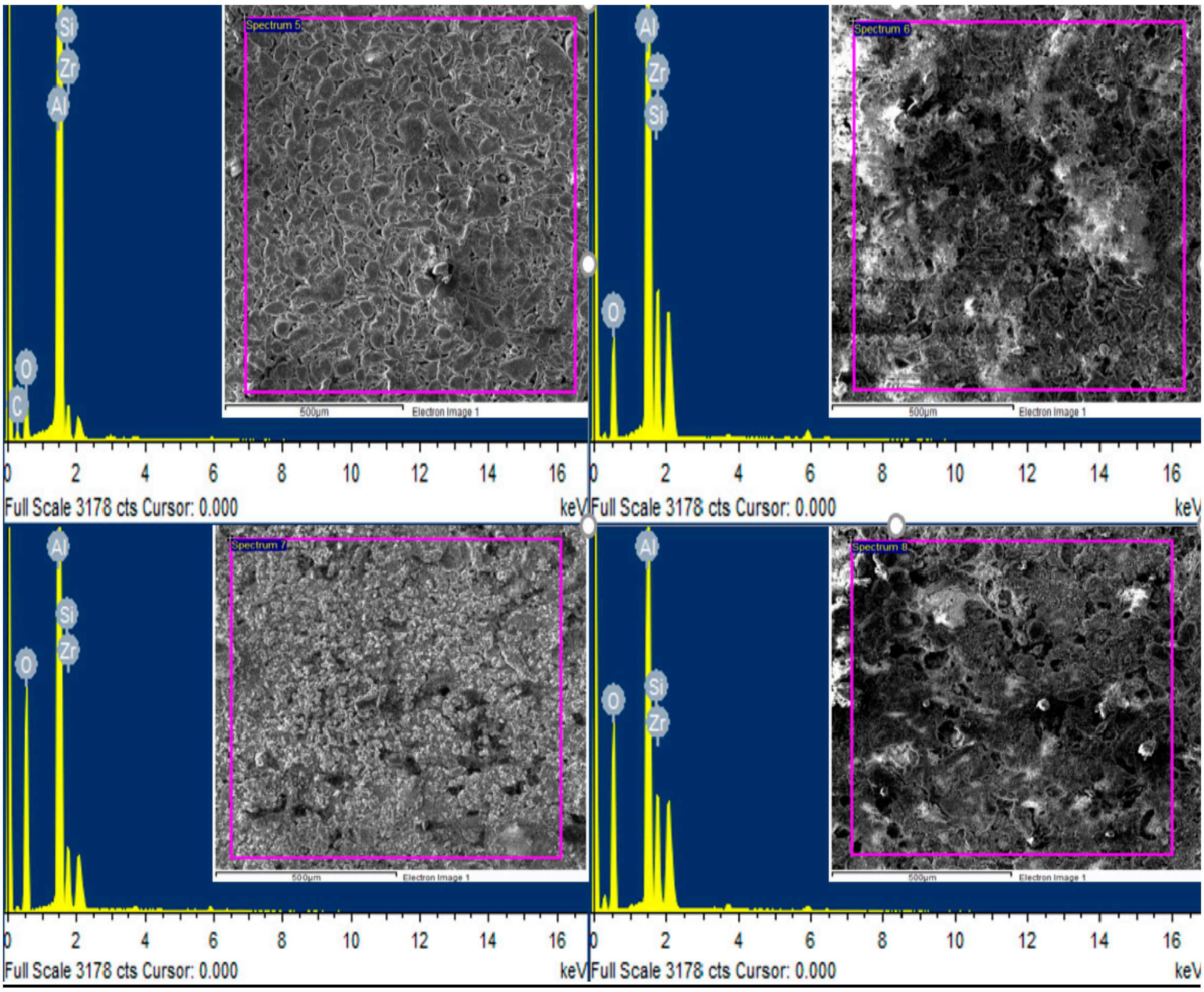

Figure S2. EDS spectra of Al- $ZrSiO_4$ reinforced binary composites with $ZrSiO_4$ reinforcement wt % of 5, 20, 30 and 40 from left to right in the row sequency.

SEM Images of hybrid Al-SiC-$ZrSiO_4$ wt% 5, 20, 30 & 40

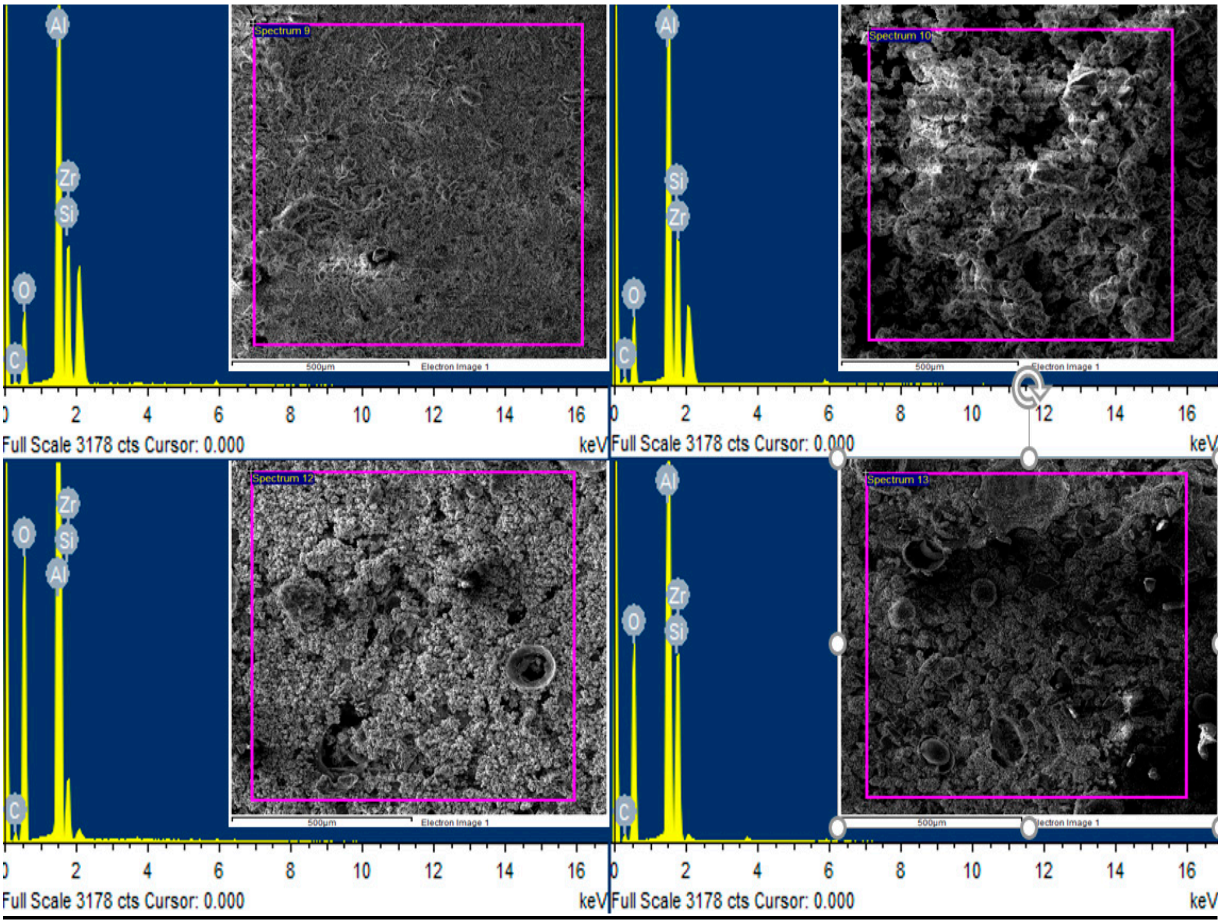

Figure S3. EDS spectra of Al-SiC-$ZrSiO_4$ hybrid reinforced hybrid composites with SiC and $ZrSiO_4$ as reinforcement with wt % of 5, 20,30 and 40.
